# Vision transformer to differentiate between benign and malignant slices in ^18^F-FDG PET/CT

**DOI:** 10.1038/s41598-024-58220-6

**Published:** 2024-04-09

**Authors:** Daiki Nishigaki, Yuki Suzuki, Tadashi Watabe, Daisuke Katayama, Hiroki Kato, Tomohiro Wataya, Kosuke Kita, Junya Sato, Noriyuki Tomiyama, Shoji Kido

**Affiliations:** 1 Department of Artificial Intelligence Diagnostic Radiology, Osaka University Graduate School of Medicine, Suita, Osaka Japan; 2 Department of Nuclear Medicine and Tracer Kinetics, Osaka University Graduate School of Medicine, Suita, Osaka Japan; 3 Department of Radiology, Osaka University Graduate School of Medicine, Suita, Osaka Japan

**Keywords:** Cancer imaging, Radionuclide imaging, Positron-emission tomography

## Abstract

Fluorine-18-fluorodeoxyglucose (^18^F-FDG) positron emission tomography (PET)/computed tomography (CT) is widely used for the detection, diagnosis, and clinical decision-making in oncological diseases. However, in daily medical practice, it is often difficult to make clinical decisions because of physiological FDG uptake or cancers with poor FDG uptake. False negative clinical diagnoses of malignant lesions are critical issues that require attention. In this study, Vision Transformer (ViT) was used to automatically classify ^18^F-FDG PET/CT slices as benign or malignant. This retrospective study included ^18^F-FDG PET/CT data of 207 (143 malignant and 64 benign) patients from a medical institute to train and test our models. The ViT model achieved an area under the receiver operating characteristic curve (AUC) of 0.90 [95% CI 0.89, 0.91], which was superior to the baseline Convolutional Neural Network (CNN) models (EfficientNet, 0.87 [95% CI 0.86, 0.88], *P* < 0.001; DenseNet, 0.87 [95% CI 0.86, 0.88], *P* < 0.001). Even when FDG uptake was low, ViT produced an AUC of 0.81 [95% CI 0.77, 0.85], which was higher than that of the CNN (DenseNet, 0.65 [95% CI 0.59, 0.70], *P* < 0.001). We demonstrated the clinical value of ViT by showing its sensitive analysis of easy-to-miss cases of oncological diseases.

## Introduction

Fluorine-18-fluorodeoxyglucose (^18^F-FDG) positron emission tomography (PET)/computed tomography (CT) is a molecular imaging technique widely used for the detection, diagnosis, and clinical decision-making for metabolically active lesions, including oncological diseases^1,2^. ^18^F-FDG uptake provides functional information on the metabolic activity of lesions and highlights where malignant tumors are present. PET/CT reliably differentiates benign tumors from malignant tumors by combining anatomical information from CT with functional information from PET^3–6^. However, in daily medical practice, it is common to have difficulty in making clinical decisions because of physiological FDG uptake or malignant lesions with poor FDG uptake^7,8^. Therefore, a wealth of specialized knowledge and experience is required to detect and differentiate between various abnormalities. This is particularly evident in the abdominopelvic region, where multiple organs exhibit physiological FDG uptake (such as the kidney, ureter, bladder, liver, intestinal tract, and adrenal gland), and where there is significant diversity in cancer origin and uptake levels. Currently, the number of experienced specialists in nuclear medicine is limited, whereas the number of PET/CT examinations is increasing^9^. As the burden on specialists in nuclear medicine increases, the risk of overlooking malignant lesions and misdiagnosis increases. Thus, there is a need for automated systems to analyze PET/CT images more efficiently.

For the automated classification of PET/CT images as benign or malignant, it is necessary to use functional information based on FDG uptake as well as anatomical information of the entire image (such as the distribution of lesions and their position relative to organs). Convolutional Neural Network (CNN) is a machine learning algorithm that has performed well in computer vision applications. However, CNN was reported to have no access to global information of the image, although it can obtain local features^10^. Sibille^11^ et al. input lesions with high FDG uptake into CNN instead of inputting the entire image. Their CNN-based system achieved high area under the receiver operating characteristic curves (AUCs) for automated cancer classification (lung cancer, 0.98; lymphoma, 0.95). However, this method has the limitation of being unable to evaluate lesions with poor FDG uptake. In clinical practice, overlooking such lesions and false negatives are critical issues, and there is a great need for a system to prevent these problems.

Vision Transformer (ViT) is an application of the transformer architecture developed for natural language processing to image classification^12,13^. The advantage of ViT compared with CNN is that it can integrate information across the entire image; ViT has the potential to outperform CNN when trained with sufficient data. Even with a small data set, it was reported that transfer learning with pretrained ViT achieved high performance^12^. ViT models pretrained on a large natural image data set, ImageNet, are publicly available^14^. Previous studies reported that fine-tuning such pretrained models for the analysis of medical images produced better performance than existing CNNs to detect COVID-19 positive cases^15,16^.

In this study, we developed and evaluated a ViT model that differentiated PET/CT slices as benign or malignant. The primary aim was to compare the performance of ViT and baseline CNN models. The secondary aim was to examine the impact of the degree of FDG activity in images on the performance of the models.

## Materials and methods

### Ethical approval

This retrospective study was approved by the institutional review boards of Osaka University Hospital (Suita, Osaka, Japan). Informed consent was waived due to the retrospective nature of the study. All procedures performed in this study involving human participants were in accordance with the ethical standards of the institutional research ethics committee and with the 1964 Helsinki declaration and its later amendments or comparable ethical standards.

### Clinical data

**Figure 1:**
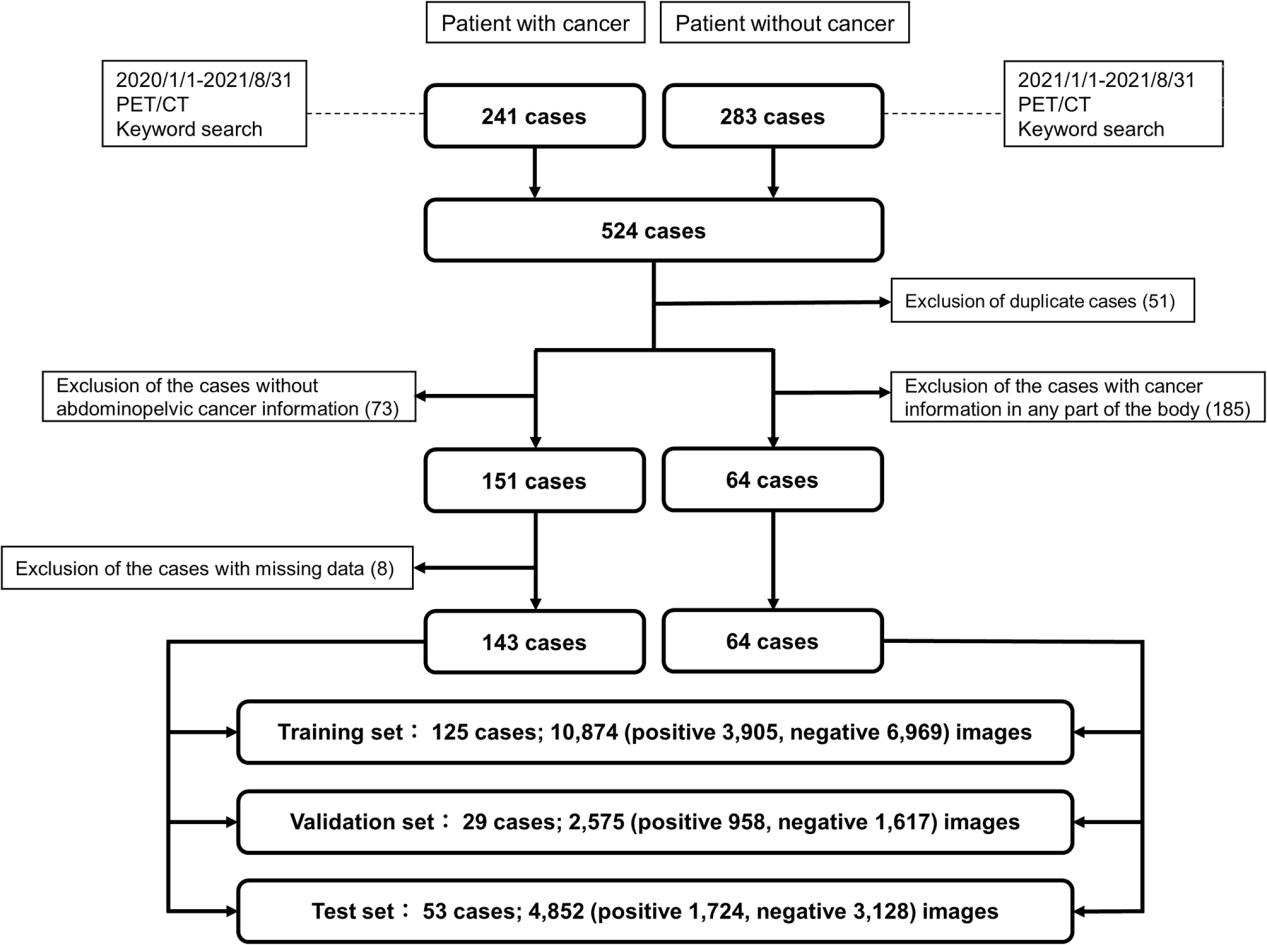
Flow chart of patient enrollment. *PET* Positron emission tomography, *CT* Computed tomography.

We retrospectively collected 143 patients with active abdominopelvic cancer and 64 patients without any active cancer, who underwent whole-body PET/CT at the Osaka University Hospital (Suita, Osaka, Japan) from January 2020 to August 2021. First, we used keyword searches in the radiology information system to collect examinations of patients with and without abdominopelvic cancer. Next, examinations other than the first one for each patient were excluded (51 cases). Third, a radiologist (D.N., 2 years of experience, in-training) inspected clinical information including radiology reports and medical records to determine the presence or absence of malignant findings. Cases without abdominopelvic cancer were excluded from the positive patient group (73 cases). Cases with malignant findings in any part of the body were excluded from the negative patient group (185 cases). Finally, cases with missing image data were excluded (8 cases). The final enrollment of 207 patients was randomly divided into training (60%), validation (15%), and test (25%) subsets while maintaining the positive-to-negative case ratio. Figure 1 shows the flowchart of patient inclusion and data partitioning.

Patients were scanned by scanner 1 (*N* = 81, Biograph Vision 600, Siemens Medical Solutions, Knoxville, TN, USA) or scanner 2 (*N* = 126, Discovery 710, GE, Milwaukee, WI, USA) at our institute. Patients fasted for six hours and were injected with 3.7 MBq per kilogram of body weight ^18^F-FDG and imaged 60 min after injection. The image acquisition details are shown in Supplementary Table 1 online.

### Data preprocessing

We acquired CT, PET, and PET/CT fusion image data in the DICOM format, which were used for clinical diagnosis and analysis and were stored at our institution. PET/CT fusion images are color images that overlay functional maps from PET onto anatomical maps from CT to facilitate the interpretation of bimodal information in clinical practice^17,18^. In our institution, PET/CT images were produced by fusing PET images with CT images at a 1:1 ratio using the Hot Iron color scale. In this study, we used axial image data of each patient’s abdominopelvic region from the diaphragm to the bladder. The flow chart of the image preprocessing is shown in Supplementary Fig. 1 online. PET and CT images were separately converted from grayscale (1 channel) to RGB (3 channels) by duplicating channels. All images were converted to 256 × 256-pixel RGB images in a similar manner, combining min–max normalization, center-cropping, connected-component labeling, resizing, and cutting margin. The preprocessing was performed using Python version 3.8.5, Pydicom version 2.1.2 (https://github.com/pydicom/pydicom), Pillow version 8.2.0 (https://github.com/python-pillow/Pillow), and OpenCV version 4.5.3 (https://github.com/opencv/opencv).

### Reference standard

Our reference standard was composed of two types of data: pathological diagnosis and image reading. The reference standard of malignant/benign diagnosis was determined according to pathological evidence when available. Lesions without histopathological diagnosis were classified through nuclear medicine expert readings. The slice-level reading-based annotation was performed using preprocessed images by two board-certified nuclear medicine experts. Images that contained potentially malignant FDG uptake (eg, primary tumor, metastases, disseminated lesions, and malignant ascites) were annotated as “positive.” Images with no suspicion (eg, no findings, physiologic uptake, bone degeneration, and inflammation) were annotated as “negative.” First, an expert (D.K., 9 years of experience) annotated all images. Then, the first annotations were double-checked by another expert (T.W., 15 years of experience). Discrepancies between annotators were resolved by consensus agreement. The experts holistically evaluated a set of three modality images (PET/CT, PET, and CT) for each slice, assigning the same label to all three images of the same slice. All clinical information, including patient background and radiology reports, was blinded for the annotators during the annotation process. In the test data, the experts manually placed bounding boxes around malignant/benign lesions as the foundation for their decisions. These bounding boxes were utilized for calculating lesion size (length of longer edge) and for qualitative evaluation of the models. Microsoft VoTT version 2.2.0 (https://github.com/microsoft/VoTT) was used for the data annotation.

### ViT model

**Figure 2:**
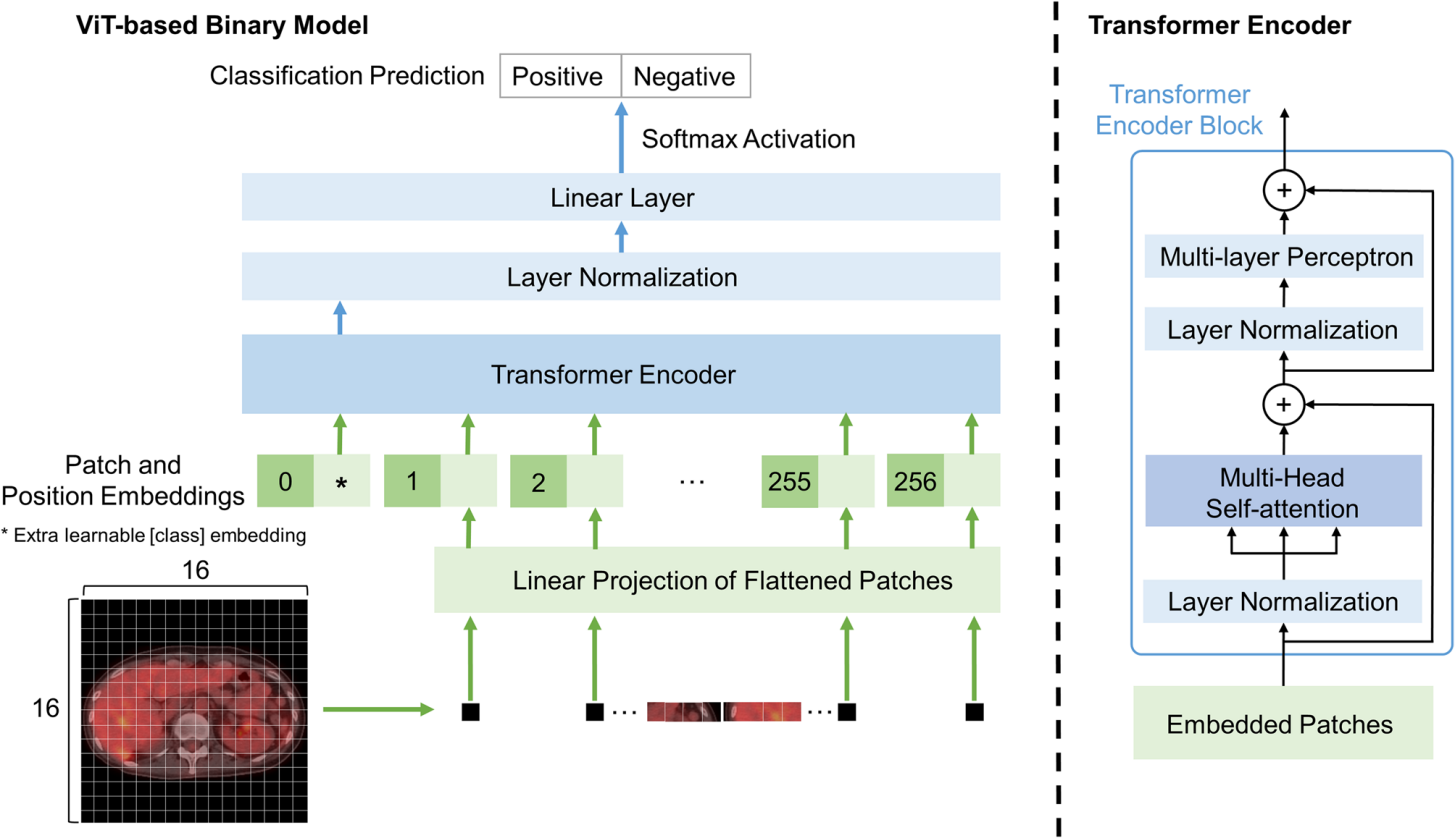
Architecture of the ViT-based binary classifier. Each image is divided into 16 × 16 patches. *ViT* Vision Transformer.

We used the ViT model to classify PET/CT images as “positive” or “negative.” We used the primary ViT model, B-16 (“Base” variant) without modification, which consists of 23 transformer encoder blocks stacked on top of each other, with a patch size of 16 × 16. The overall architecture is shown in Fig. 2, and the network architecture details are as follows. Because ViT treats image data as a sequence of small patches, the initial part of the network has a patch encoder layer that reshapes the input image into multiple flattened patches. Next, position embeddings are added to the patches to preserve the structural and neighborhood information. The sequence is then appended with the [class] embedding and input to the transformer encoder. The transformer encoder is the same as that of Vaswani et al.^13^, which contains multi-headed self-attention layers and multiple multi-layer perceptron blocks. Layer normalization is used before each block, which assists in reducing training time and improving generalization performance. The transformer encoder outputs feature vectors corresponding to the input patches. Following the standard method, we used the first feature vector corresponding to the [class] embedding, which represents the entire sequence. Finally, a learnable linear layer processes this feature vector and outputs a binary vector, followed by softmax activation.

### CNN models

ViT was compared with two baseline CNN models, DenseNet and EfficientNet. DenseNet is a CNN composed of DenseBlocks. DenseBlocks allows convolutional networks to learn more deeply, accurately, and efficiently than conventional convolutional layers do by connecting each layer to the others in a feed-forward fashion, achieving high performance while reducing memory and computation. We used DenseNet-121, one type of DenseNet, that has proven effective at medical image classification^19,20^. EfficientNet is a model that has achieved state-of-the-art capabilities on various benchmark datasets while significantly reducing computational costs for image recognition, utilizing a composite scaling method to enlarge network depth, width, and resolution^21^. EfficientNet has been used for the classification of many medical images and its good performance has been demonstrated^22–24^. There are eight types of base models from B0 to B7, and each model has a different expected input shape. Considering an input image size of 256 × 256 pixels, EfficientNet-B0, -B1, and -B2 were selected.

### Training method and determining optimal hyperparameters

Image-wise fine-tuning was performed on ViT, EfficientNet, and DenseNet models that were pretrained on ImageNet-21k^14^. We removed the pretrained linear (classification) layers and attached a new learnable linear layer. Fine-tuning was performed for all layers, including the pretrained layers. All training images were input in random order, and not separated by case. Grid search was performed to determine optimal hyperparameters from the set of candidates shown in Supplementary Table 2 online using the accuracy on the validation set. The optimal batch size was 8 and learning rate was 1e^-3^ across all models. Drop rates were determined according to the original study of each model (ViT, 0.1; EfficientNet, 0.2; DenseNet, 0.2)^12,19,21^. We used the stochastic gradient descent with momentum (momentum = 0.9) and cross-entropy loss function. The models were respectively fine-tuned for sufficient numbers of epochs to converge the validation accuracies, and weights with the lowest validation losses were used for the test. The learning environment was an NVIDIA Titan RTX graphics processing unit and CUDA version 10.1 (https://developer.nvidia.com/cuda-10.1-download-archive-base). Our systems were written entirely in Python version 3.8.5. We used PyTorch version 1.8.1, scikit-learn version 0.24.2 (https://scikit-learn.org), timm version 0.4.12 (https://github.com/rwightman/pytorch-image-models), and pytorch-pretrained-vit version 0.0.7 (https://github.com/lukemelas/PyTorch-Pretrained-ViT) to build our models. We calculated the evaluation metrics using NumPy version 1.21.2 and scikit-learn version 0.24.2.

### Comparison of performance between ViT and CNN models

We compared the classification performance between ViT, EfficientNet, and DenseNet on 4,852 test PET/CT images. Each model outputs a probability of malignancy for each input image. We calculated the AUC as the performance metric. We also performed qualitative evaluation by visualizing important regions that contributed to the prediction of each model. The Gradient-weighted Class Activation Mappings (Grad-CAMs) of all models were compared. Grad-CAM is a visualization method that uses gradient information^25^. We computed importance scores from the gradient information for each class (“positive”/ “negative”) flowing into the final transformer block or convolution layer. We set the cutoff point using the Youden Index in the validation data and converted each probability into a binary prediction of “positive”/“negative.” Important regions were highlighted according to the importance score of each pixel in the input image. After min–max normalization, the score matrix of each image was converted into a heat map for visualization.

### Evaluation of the influence of input image modality on classification performance

When functional information in PET is insufficient to make a diagnosis, anatomical information in CT helps readers to better understand the lesion; therefore, bimodal analysis using PET and CT images is crucial for a diagnosis by PET/CT. To assess the role of each modality in PET/CT diagnosis, we trained and evaluated two additional ViT models, each of which only utilized either PET or CT inputs. We used the same fine-tuning configuration described in the “Training Method” section except for the input modalities. The performance of the three fine-tuned ViT models was evaluated using the test set of each modality. Qualitative evaluation was also performed by comparing the Grad-CAMs of each model.

### Statistical analysis

For statistical comparisons, the AUCs and the 95% confidence intervals (CIs) were computed and compared using the DeLong test. Statistical significance was indicated by *P* values < 0.05. Bonferroni correction was used for multiple comparisons. R version 4.1.2 and pROC package version 1.18 were used for statistical computations^26^.

## Results

### Patient characteristics and distribution of annotated image data

Overall, 143 patients (mean age, 65 ± 16 [SD] years; 54 males) had cancer, and 64 patients (mean age, 64 ± 17 years; 39 males) did not have cancer. Pathological diagnoses of study patients are presented in Table 1. Full details of patient characteristics, including primary cancer type, stage at initial diagnosis, and previous therapy, are summarized in Supplementary Table 3 online. Supplementary Table 4 online shows the distribution of the annotated image data: 6,587 (36.0%) of 18,301 PET/CT images and 6,575 (35.9%) of 18,302 PET or CT images were labeled “positive.” The average lesion size was 95.1 ± 66.9 mm for malignant lesions and 60.0 ± 17.9 mm for benign lesions in the test data. The average size of the focal malignant lesions (excluding diffuse lesions such as malignant ascites) was 71.5 ± 33.8 mm.

**Table 1:** Pathological diagnoses of lesions in study patients for which histological examinations were performed.

Pathological diagnosis	Number
Malignant (Cancer)	
Stomach	12
Colon and rectum	22
Liver	4
Bile ducts	4
Gallbladder	2
Ampulla of vater	1
Pancreas	17
Soft tissues	3
Gastrointestinal stromal tumor	4
Cervix uteri	6
Uterus—Endometrium	7
Ovarian, fallopian tube, and primary peritoneal carcinoma	18
Lymphoma, and other hematological malignancy	2
Pediatric tumors	1
Benign	
Colorectal polfyp	6

### Comparison of performance between ViT and CNN models

**Figure 3:**
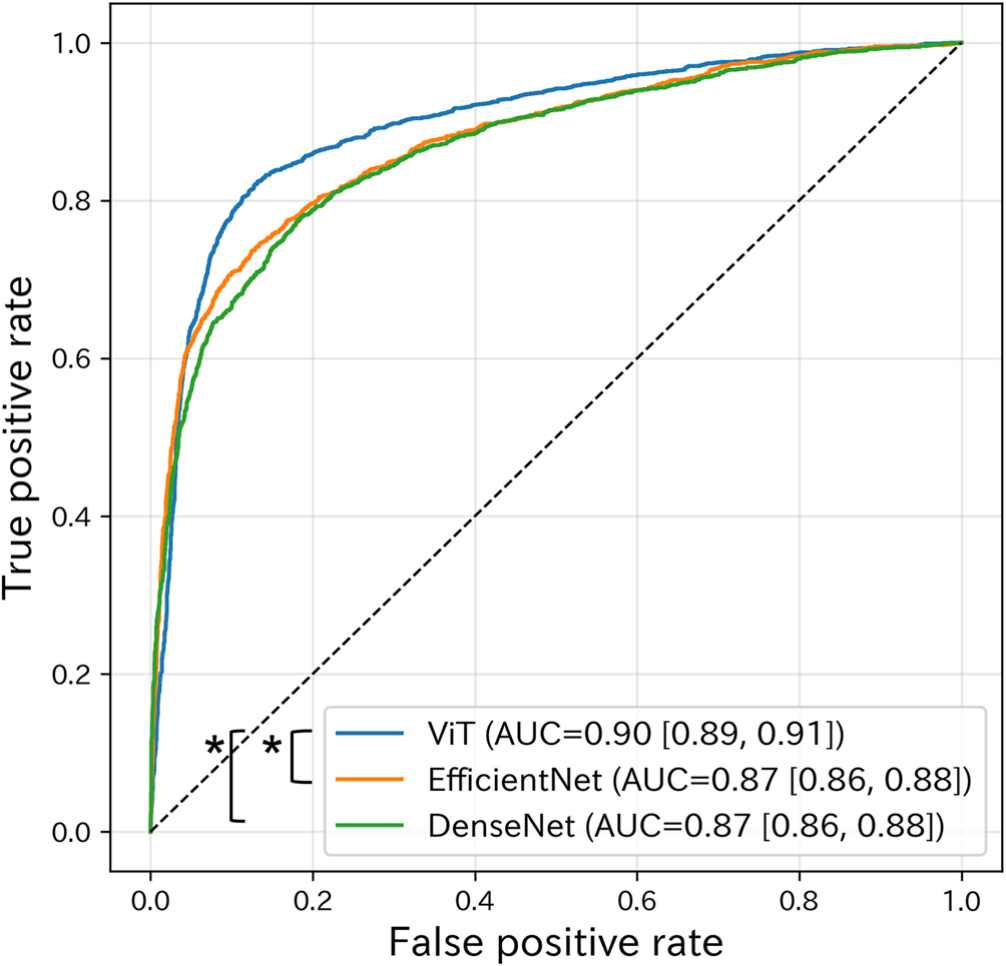
Receiver operating characteristic curves of ViT, EfficientNet, and DenseNet models. Data in square brackets are 95% confidence intervals. “*” represents *P* < 0.001. *ViT* Vision Transformer, *AUC* Area under the receiver operating characteristic curve.

Figure 3 represents the receiver operating characteristic (ROC) curves for ViT, EfficientNet, and DenseNet models. For EfficientNet, the B0 model produced the highest validation accuracy (0.872) in the EfficientNet models (B1, 0.864; B2, 0.866) and was used for further analyses. ViT achieved an AUC of 0.90 [95% CI 0.89, 0.91], which was higher than that of EfficientNet (0.87 [95% CI 0.86, 0.88]; *P* < 0.001) and DenseNet (0.87 [95% CI 0.86, 0.88]; *P* < 0.001). ViT and EfficientNet fine-tuned models outperformed each respective from-scratch model, with no significant differences found for DenseNet (Supplementary Table 5 online); therefore, we only showed the results of the fine-tuned models. We computed slice-level maximum standardized uptake values (SUVmax) for stratified analyses (Table 2). When FDG uptake was unremarkable (SUVmax ≤ 7.0), ViT had higher AUCs (SUVmax 3.5–7.0, 0.88 [95% CI 0.86, 0.90]; SUVmax < 3.5, 0.81 [95% CI 0.77, 0.85]) compared with EfficientNet (SUVmax 3.5–7.0, 0.85 [95% CI 0.83, 0.87], *P* < 0.001) and DenseNet (SUVmax 3.5–7.0, 0.82 [95% CI 0.80, 0.84], *P* < 0.001; SUVmax < 3.5, 0.65 [95% CI 0.59, 0.70], *P* < 0.001). Supplementary Fig. 2 online shows accuracy and loss curves of the ViT, DenseNet, and EfficientNet. The average training time per epoch for EfficientNet was the shortest among the models (EfficientNet, 102 s; DenseNet, 167 s; ViT, 269 s). All pretrained models tended to overfit as the training progressed.

**Table 2:** Stratified analysis by the SUVmax of AUCs of ViT, EfficientNet, and DenseNet models on the test set.

		ViT	EfficientNet		DenseNet	
	Percentage of positive images	AUC	AUC	*P* value (vs. ViT)	AUC	*P* value (vs. ViT)
All	35.5% (1724/4852)	0.90 [0.89, 0.91]	0.87 [0.86, 0.88]	*P* < 0.001	0.87 [0.86, 0.88]	*P* < 0.001
SUVmax > 7.0	50.8% (989/1946)	0.89 [0.87, 0.90]	0.88 [0.86, 0.89]	*P* = 0.100	0.91 [0.90, 0.92]	*P* = 0.004
SUVmax 3.5–7.0	40.9% (610/1490)	0.88 [0.86, 0.90]	0.85 [0.83, 0.87]	*P* < 0.001	0.82 [0.80, 0.84]	*P* < 0.001
SUVmax < 3.5	8.8% (125/1416)	0.81 [0.77, 0.85]	0.77 [0.72, 0.81]	*P* = 0.094	0.65 [0.59, 0.70]	*P* < 0.001

Data in parentheses are numerators/denominators for percentages. Data in square brackets are 95% confidence intervals. *AUC* Area under the receiver operating characteristic curve, *SUVmax* Maximum standardized uptake value, *ViT* Vision Transformer.

### Comparison of heatmaps between models

**Figure 4:**
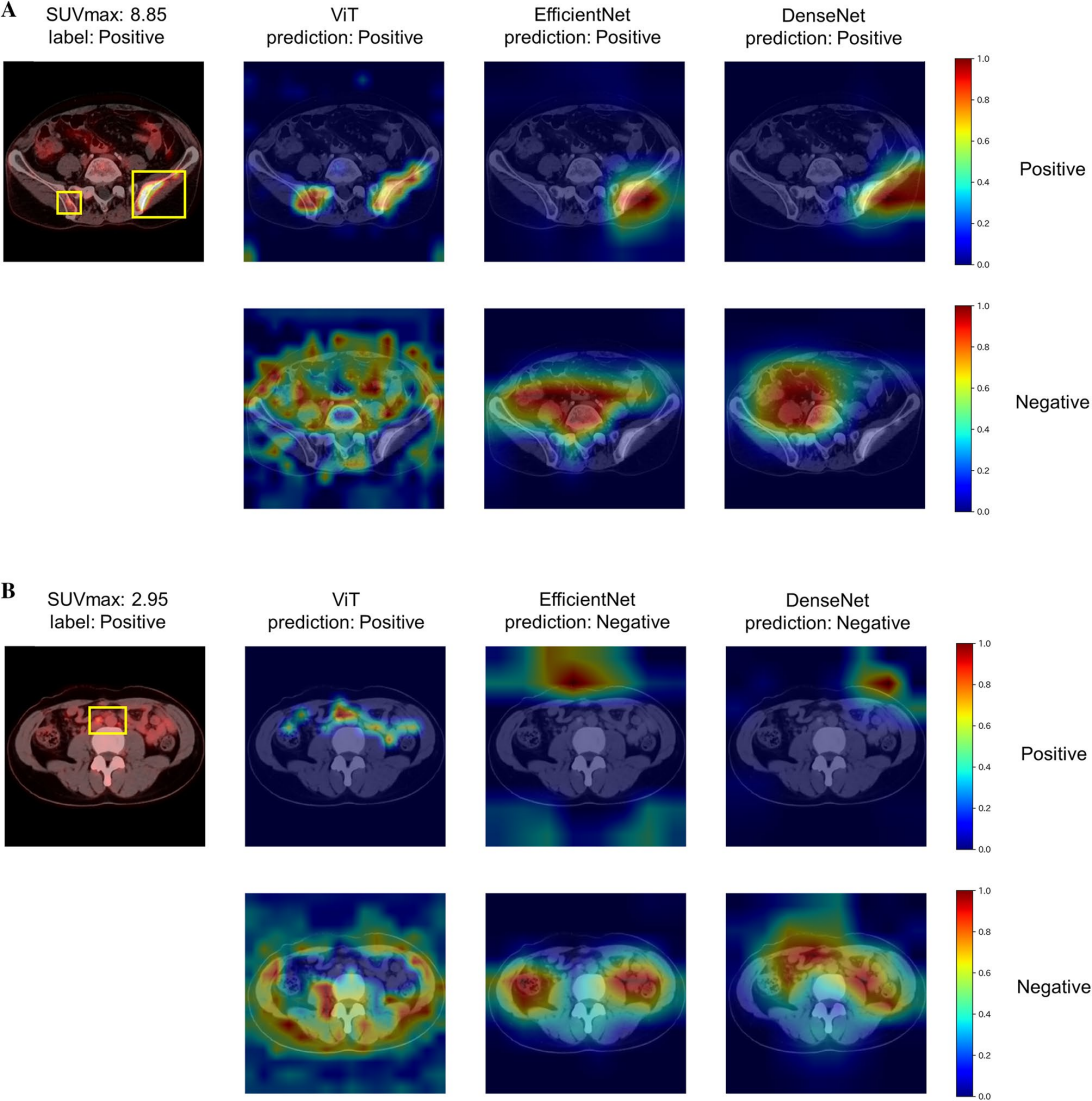

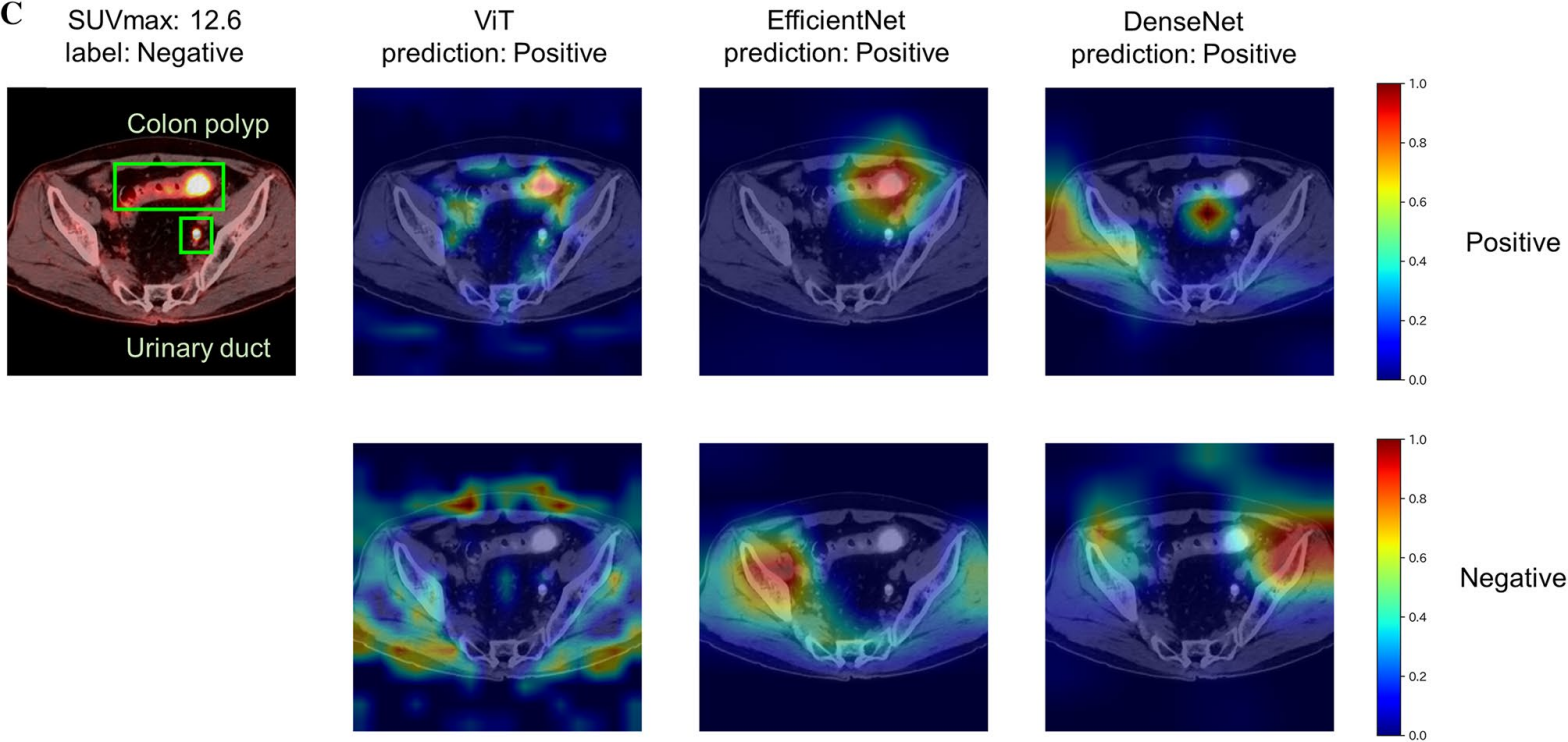
Predictions and Grad-CAMs of ViT, EfficientNet, and DenseNet models on sample test images. The yellow bounding boxes indicate malignant lesions and the green bounding boxes present benign lesions. The top rows of the Grad-CAMs show important areas for a “positive” prediction, and the bottom rows show areas for a “negative” prediction. *SUVmax* Maximum standardized uptake value, *ViT* Vision Transformer, *Grad-CAM* Gradient-weighted class activation mapping.

Figure 4 shows the predictions and Grad-CAMs of ViT, EfficientNet, and DenseNet on sample test images from the “positive” class. In example (a), the predictions of all models were correct and ViT recognized bone metastasis in the left and right ilium. However, CNN focused only on the left lesion. In sample (b), ViT focused on lymphadenopathy with faint FDG uptake (SUVmax: 2.95), whereas CNNs failed to capture that region. An example of false positive prediction is shown in Fig. 4C. No model was able to predict “negative” for a lesion that was suspected to be malignant in PET/CT diagnosis but histologically diagnosed as a colon polyp.

### Evaluation of the influence of input image modality on classification performance

**Figure 5:**
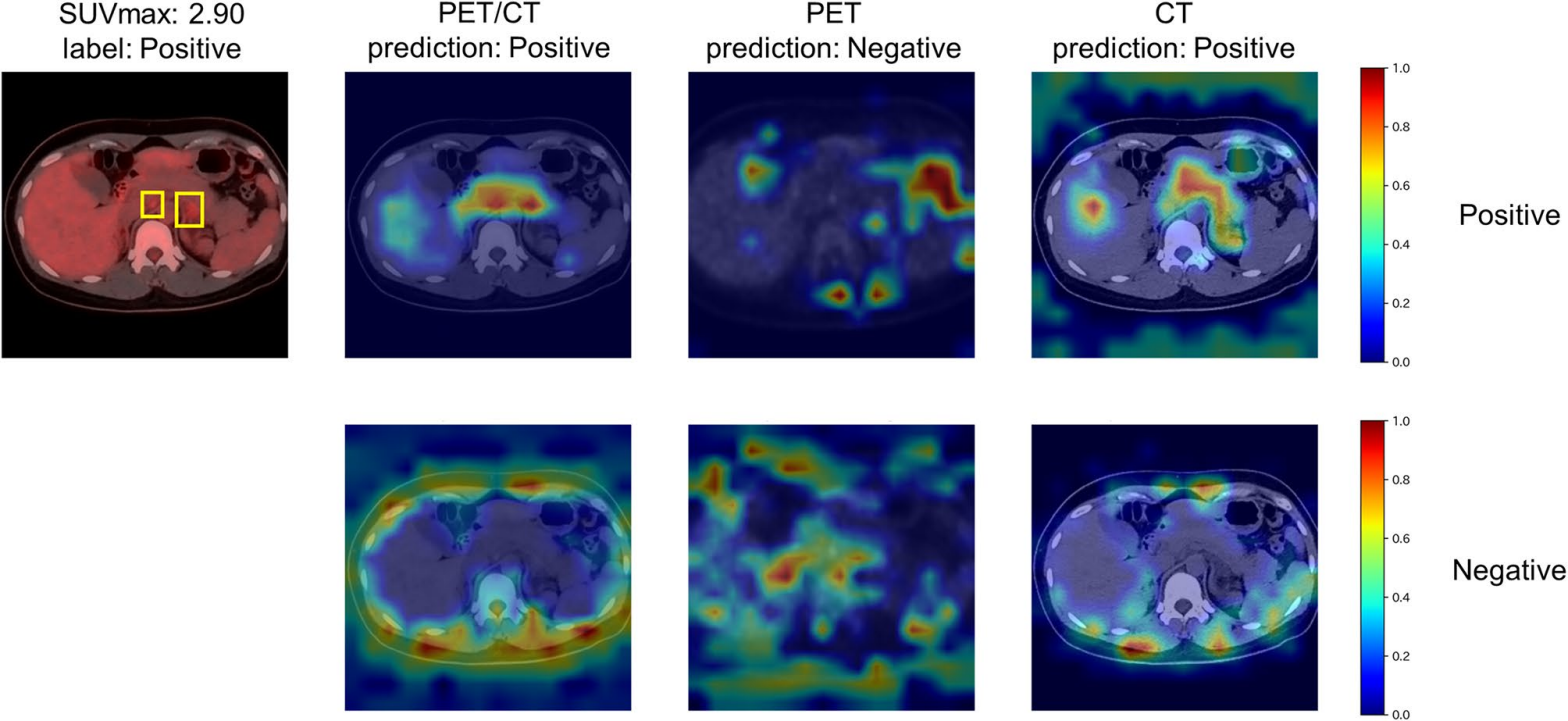
Predictions and Grad-CAMs of ViT-based models on sample PET/CT, PET, and CT test images from the “positive” class. ViT was fine-tuned using training data of each modality. The bounding boxes depicted in the figure indicate malignant lesions. The top row of the Grad-CAMs shows important areas for “positive” predictions, and the bottom row shows areas for “negative” predictions. *PET* Positron emission tomography, *CT* Computed tomography, *SUVmax* Maximum standardized uptake value, *ViT* Vision Transformer, *Grad-CAM* Gradient-weighted class activation mapping.

The AUCs of the ViT-based models with different input types are shown in Table 3. PET/CT fusion input had higher AUCs than CT input across all SUVmax levels. No evidence of difference was found between the PET/CT and PET input for the whole data analysis (the All row). However, when FDG uptake was low (SUVmax < 3.5), the AUC of ViT using PET/CT (0.81 [95% CI 0.77, 0.85]) was higher than using PET data alone (0.61 [95% CI 0.55, 0.67]; *P* < 0.001) by a large margin. An example of a “positive” class slice with low FDG uptake (SUVmax: 2.90) is shown in Fig. 5, where the predictions and Grad-CAMs of ViT models for PET/CT, PET, and CT inputs are compared. In the example, ViT recognized lymphadenopathy and predicted “positive” using the PET/CT image, but it failed to detect the lesion in the PET image.

**Table 3:** Stratified analysis by the SUVmax of AUCs of ViT models on the PET/CT, PET, and CT test set.

		PET/CT	PET		CT	
	Percentage of positive images	AUC	AUC	*P* value (vs. PET/CT)	AUC	*P* value (vs. PET/CT)
All	35.5% (1724/4852)	0.90 [0.89, 0.91]	0.88 [0.87, 0.90]	*P* = 0.029	0.70 [0.69, 0.72]	*P* < 0.001
SUVmax > 7.0	50.8% (989/1946)	0.89 [0.87, 0.90]	0.93 [0.92, 0.94]	*P* < 0.001	0.71 [0.69, 0.73]	*P* < 0.001
SUVmax 3.5–7.0	40.9% (610/1490)	0.88 [0.86, 0.90]	0.86 [0.84, 0.88]	*P* = 0.033	0.74 [0.70, 0.76]	*P* < 0.001
SUVmax < 3.5	8.8% (125/1416)	0.81 [0.77, 0.85]	0.61 [0.55, 0.67]	*P* < 0.001	0.67 [0.61, 0.73]	*P* < 0.001

ViT was fine-tuned using training data of each modality. Data in parentheses are numerators/denominators for percentages. Data in square brackets are 95% confidence intervals. *AUC* Area under the receiver operating characteristic curve, *PET* Positron emission tomography, *CT* Computed tomography, *SUVmax* Maximum standardized uptake value, *ViT* Vision Transformer.

## Discussion

In this study, we developed a ViT-based system to automatically differentiate ^18^F-FDG uptake of PET/CT at the slice level. The ViT model achieved an AUC of 0.90, which was superior to the CNN models for the classification of PET/CT slices as benign or malignant. Even when the FDG uptake was low (SUVmax < 3.5), ViT produced an AUC of 0.81, which was higher than that of the CNNs. This demonstrated the usefulness of ViT for classifying FDG uptake from PET/CT images.

Previous studies demonstrated the efficacy of utilizing deep learning in the classification of ^18^F-FDG PET/CT images as benign or malignant. Sibille et al. achieved a AUC of 0.98 in the classification of lesions with high FDG uptake in lung cancer and lymphoma patients^11^. Häggström et al. attained an AUC of 0.939 in the classification of 3D PET/CT images of lymphoma patients^27^. However, these studies focused on specific diseases, which limits their applicability to the variety of lesions encountered in clinical practice. Eyuboglu et al. developed a deep learning model for cross-disease abnormality detection at the organ level using weak supervision. Their model achieved a mean AUC exceeding 0.85 in 10 regions, including lungs, liver, and thoracic lymph nodes^28^. Their study was primarily aimed at detecting abnormal metabolic activity and no classification of the identified lesions as benign or malignant was performed. In our study, patients with various diseases were included and ViT achieved an AUC of 0.90 in differentiating PET/CT images (with histopathological evidence for some lesions). This is more valuable in clinical practice compared to previous studies.

We demonstrated that the ViT model had significantly higher performance than the CNN models for the classification of PET/CT images. Previous research suggested that a ViT model with architecture similar to ViT-B16 had no evident performance advantage over DenseNet-121 for the classification of radiological images^29,30^. They performed a diagnosis of disease on chest radiographs (eg, atelectasis, cardiomegaly, and effusion) and extremity radiographs (eg, bone fracture and amputation). Differentiating PET/CT images as benign or malignant is a more complex task than using X-ray images because it requires the integration of functional information from PET and anatomical information from CT (such as the distribution of lesions and their position relative to organs) from the entire image. ViT was superior to existing models in differentiating PET/CT images as benign or malignant because ViT can integrate information across the entire image better than CNN can^12^. Figure 4A showed that ViT tended to identify lesions more accurately than CNNs, which was consistent with the previous report^30^. In some cases, however, ViT made predictions that diverged from pathological diagnoses (Fig. 4C). Increasing the number of training data linked to histopathological evidence will reduce such false positives and false negatives, avoiding unnecessary additional tests and improving patient prognosis. We showed that ViT had a high performance when the FDG uptake was unremarkable in PET/CT images as shown in Table 2 and Fig. 4B, whereas its performance was reduced when using PET information only. This indicates ViT can leverage anatomical information from CT images to disambiguate subtle FDG uptake in PET images.

The effects of hidden stratification can be problematic in machine learning for medical imaging^31^. Previous research using CNN to identify pneumothorax in chest radiographs reported it was affected by hidden stratification where the presence of pneumothorax correlated with the presence of chest tubes that were placed for its treatment. CNN trained to identify pneumothorax in X-ray images had a higher AUC on images with chest tubes than on images without chest tubes, and Grad-CAM indicated that the CNN focused on chest tubes. These previous studies highlighted the potential limitation of machine learning algorithms where classifiers can be fixated on salient features (chest tubes), and overlook clinically significant features (eg, collapsed lungs)^32,33^. A shortcut for the classification of FDG uptake in PET/CT images as benign or malignant is to classify strong FDG uptake as malignant. Thus, there is a concern that models will be trained to focus on regions with high FDG uptake and to undervalue lesions with poor FDG uptake. Our stratified analysis showed that the ViT model achieved higher AUCs when the FDG uptake was unremarkable and recognized lesions with low FDG uptake compared with the CNN models. This suggests that ViT is less susceptible to hidden stratification than CNN, in accordance with previous studies^30^. Our results indicated that ViT might be used to address the problem of potential confounding features in medical imaging datasets used for machine learning.

Our study had limitations. First, we used data from a single institution. Our system may have overfitted data to the epidemiology specific to that hospital. Second, we used a single type of color scale for PET/CT fusion images. The color scale of fusion images may vary by facility. Extending this study to other institutions with different color scales for PET/CT fusion images is an important future task. Third, not all lesions in our reference standard data have histopathological evidence. There may be a discrepancy between imaging diagnosis and pathological diagnosis. Finally, the set of candidates of hyperparameters was limited (see Supplementary Table 2 and Supplementary Fig. 2 for details) and we seek to investigate better optimal parameters for our models in the future.

In conclusion, we demonstrated that the ViT model performed better than the CNN models for the classification of PET/CT slices as benign or malignant. The ViT model retained a relatively high AUC for input slices with a low SUVmax, which demonstrated the clinical value of ViT related to its sensitivity to easy-to-miss cases. We expect that the ViT model will help users to differentiate between benign and malignant slices in PET/CT images and prevent overlooking lesions with insignificant FDG uptake.

### Supplementary Information

Supplementary Information.

## Data Availability

All clinical information and PET/CT image data are limitedly available through formal approval procedures upon requests to validated investigators. Further requests and inquiries are available to corresponding author (S.K.).
