# Supplementary material for: Vision transformer to differentiate between benign and malignant slices in 18F-FDG PET/CT
Source: Sci Rep. 2024 Apr 9;14:8334. doi: 10.1038/s41598-024-58220-6 (PMC11004130; doi:10.1038/s41598-024-58220-6)
Supplement: Supplementary file 1 — Supplementary Information. [file 41598_2024_58220_MOESM1_ESM.pdf]

	Biograph Vision 600 (Siemens)		Discovery 710 (GE)	
	PET	CT	PET	CT
Matrix size	440	512	192	512
Voxel spacing	1.65 × 1.65 × 3.0	0.98 × 0.98 × 3.0	3.65 × 3.65 × 3.27	0.98 × 0.98 × 3.75
Gaussian post filter	3	—	4	—
Reconstruction method	point spread function and TOF	—	OSEM, point spread function, and TOF	—
kVp	—	120	—	120
mA	—	Auto [noise index = 15]	—	CARE Dose4D [Quality ref. mAs = 100 mA]
Reconstruction Kernel	—	standard	—	standard
Slice thickness	3.0	3.0	3.27	3.75
Slice interval	3.0	3.0	3.27	3.27
Windowing	SUV 0–7 g/ml	center 40 HU, width 300 HU	SUV 0–7 g/ml	center 40 HU, width 400 HU

Supplementary Table 1. Image acquisition details.

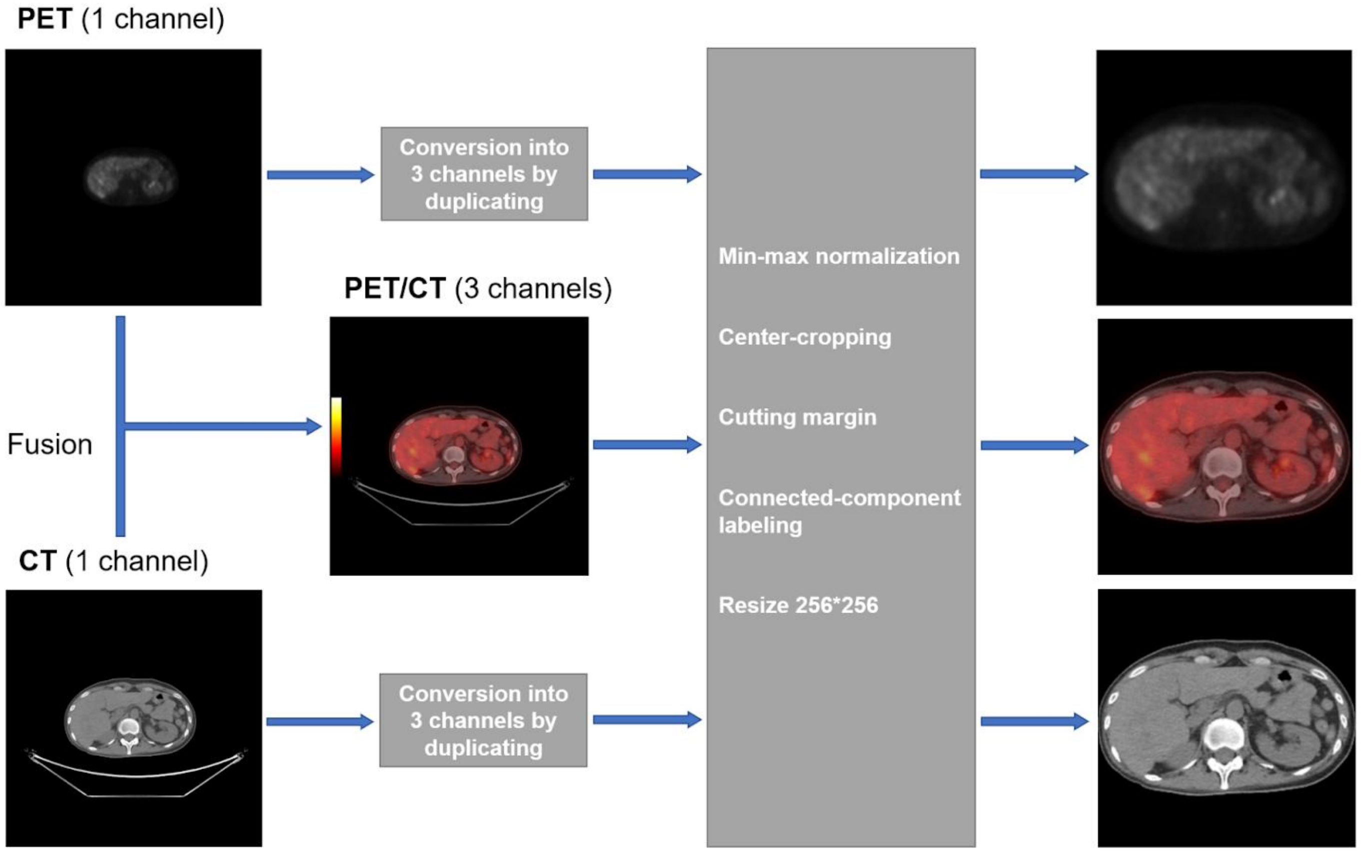

Supplementary Figure 1. Flow chart of image preprocessing, in which images were preprocessed to remove irrelevant areas for diagnosis (e.g., beds and air). PET, positron emission tomography; CT, computed tomography; SUV, standardized uptake value; WC, window center: WW, window width.

Model	Hyperparameter	Candidate values	Final value
ViT	Batch size	4, 8	8
	Learning rate	1e-3, 1e-4	1e-3
EfficientNet	Batch size	4, 8	8
	Learning rate	1e-3, 1e-4	1e-3
DenseNet	Batch size	4, 8	8
	Learning rate	1e-3, 1e-4	1e-3

Supplementary Table 2. Hyperparameters in the training of each machine learning model. The optimal hyperparameters were determined by grid search. ViT, Vision Transformer.

	Patient with cancer	Patient without cancer
No. of Patients	143	64
Sex		
Male	54 (37.8)	39 (60.9)
Female	89 (62.2)	25 (39.1)
Mean age (year)	65 ± 16	64 ± 17
Primary Cancer Type		
Pharynx	3	
Larynx	2	
Nasal Cavity and Paranasal Sinuses	1	2
Major Salivary Glands	4	
Esophagus	4	5
Stomach	12	
Colon and Rectum	17	1
Liver	4	
Bile Ducts	4	
Gallbladder	2	
Ampulla of Vater	1	
Pancreas	17	
Lung	9	2
Thymic Tumors	3	
Soft Tissues	3	
Gastrointestinal Stromal Tumor	4	
Breast	13	
Cervix Uteri	6	
Uterus - Endometrium	7	
Ovarian, Fallopian Tube, and Primary Peritoneal Carcinoma	19	
Prostate		1
Lymphoma, and other Hematological Malignancy	2	40

Pediatric Tumors	1	
Unknown primary	3	
Stage at initial diagnosis		
Ⅰ(%)	9	4
Ⅱ(%)	21	10
Ⅲ(%)	40	19
Ⅳ(%)	14	14
Status at examinations		
At initial diagnosis	64 (44.8)	13 (20.3)
During therapy	35 (24.5)	11 (17.2)
After therapy	44 (30.8)	40 (62.5)
Therapy		
Operation (%)	46	11
Chemotherapy (%)	45	73
Radiation therapy (%)	13	14

Supplementary Table 3. Clinical characteristics of study patients and indications for ^{18}F-FDG PET/CT imaging. Data in parentheses are percentages. Mean data include ± SD. FDG, fluorodeoxyglucose; PET, positron emission tomography; CT, computed tomography.

	All	Positive		Negative	
		Siemens	GE	Siemens	GE
No. of PET/CT images	18301	3259 (17.8)	3328 (18.2)	4238 (23.2)	7476 (40.9)
No. of PET images	18302	3256 (17.8)	3319 (18.1)	4241 (23.2)	7486 (40.9)
No. of CT images	18302	3256 (17.8)	3319 (18.1)	4241 (23.2)	7486 (40.9)

Supplementary Table 4. Distribution of the annotated image data. Data in parentheses are percentages. PET, positron emission tomography; CT, computed tomography.

Model		AUC	*P* value vs. From-scratch
ViT	Fine-tuned	**0.90** (0.89, 0.91)	$P < .001$
	From-scratch	0.72 (0.70, 0.73)	—
EfficientNet	Fine-tuned	**0.87** (0.86, 0.88)	$P < .001$
	From-scratch	0.81 (0.80, 0.83)	–
DenseNet	Fine-tuned	0.87 (0.86, 0.88)	$P = .053$
	From-scratch	0.85 (0.84, 0.87)	–

Supplementary Table 5. AUCs and statistical analysis results of fine-tuned and from-scratch models on the test set. Data in parentheses are 95% confidence intervals. AUC, area under the receiver operating characteristic curve; ViT, Vision Transformer.

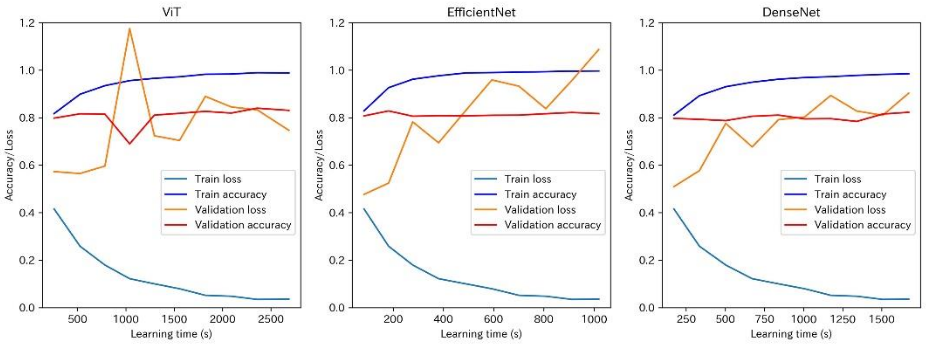

Supplementary Figure 2. Accuracy and loss curves of Vision Transformer, DenseNet, and EfficientNet.
